# Hordatines as a Potential Inhibitor of COVID-19 Main Protease and RNA Polymerase: An In-Silico Approach

**DOI:** 10.1007/s13659-020-00275-9

**Published:** 2020-10-22

**Authors:** Mohammed A. Dahab, Mostafa M. Hegazy, Hatem S. Abbass

**Affiliations:** 1 Department of Pharmaceutical Medicinal Chemistry and Drug Design, Faculty of Pharmacy, Al-Azhar University (Boys), Cairo, 11884 Egypt; 2 Department of Pharmacognosy, Faculty of Pharmacy, Al-Azhar University (Boys), Cairo, 11884 Egypt; 3 Department of Pharmacognosy, Faculty of Pharmacy, Sinai University, Kantara, 41636 Egypt

**Keywords:** Barley, COVID-19, Docking, Hordatine, Protease, RNA polymerase, MOE

## Abstract

**Abstract:**

Total 40 natural compounds were selected to perform the molecular docking studies to screen and identify the potent antiviral agents specifically for Severe Acute Respiratory Syndrome Coronavirus 2 that causes coronavirus disease 2019 (COVID-19). The key targets of COVID-19, protease (PDB ID: 7BQY) and RNA polymerase (PDB ID: 7bV2) were used to dock our target compounds by Molecular Operating Environment (MOE) version 2014.09. We used 3 different conformations of protease target (6M0K, 6Y2F and 7BQY) and two different score functions to strengthen the probability of inhibitors discovery. After an extensive screening analysis, 20 compounds exhibit good binding affinities to one or both COVID-19 targets. 7 out of 20 compounds were predicted to overcome the activity of both targets. The top 7 hits are, flacourticin **(3)**, sagerinic acid **(16)**, hordatine A **(23)**, hordatine B **(24)**, *N*-feruloyl tyramine dimer **(25)**, bisavenanthramides B-5 **(29)** and vulnibactins **(40)**. According to our results, all these top hits was found to have a better binding scores than remdesivir, the native ligand in RNA polymerase target (PDB ID: 7bV2). Hordatines are phenolic compounds present in barley, were found to exhibit the highest binding affinity to both protease and polymerase through forming strong hydrogen bonds with the catalytic residues, as well as significant interactions with other receptor-binding residues. These results probably provided an excellent lead candidate for the development of therapeutic drugs against COVID-19. Eventually, animal experiment and accurate clinical trials are needed to confirm the preventive potentials of these compounds.

**Graphic Abstract:**

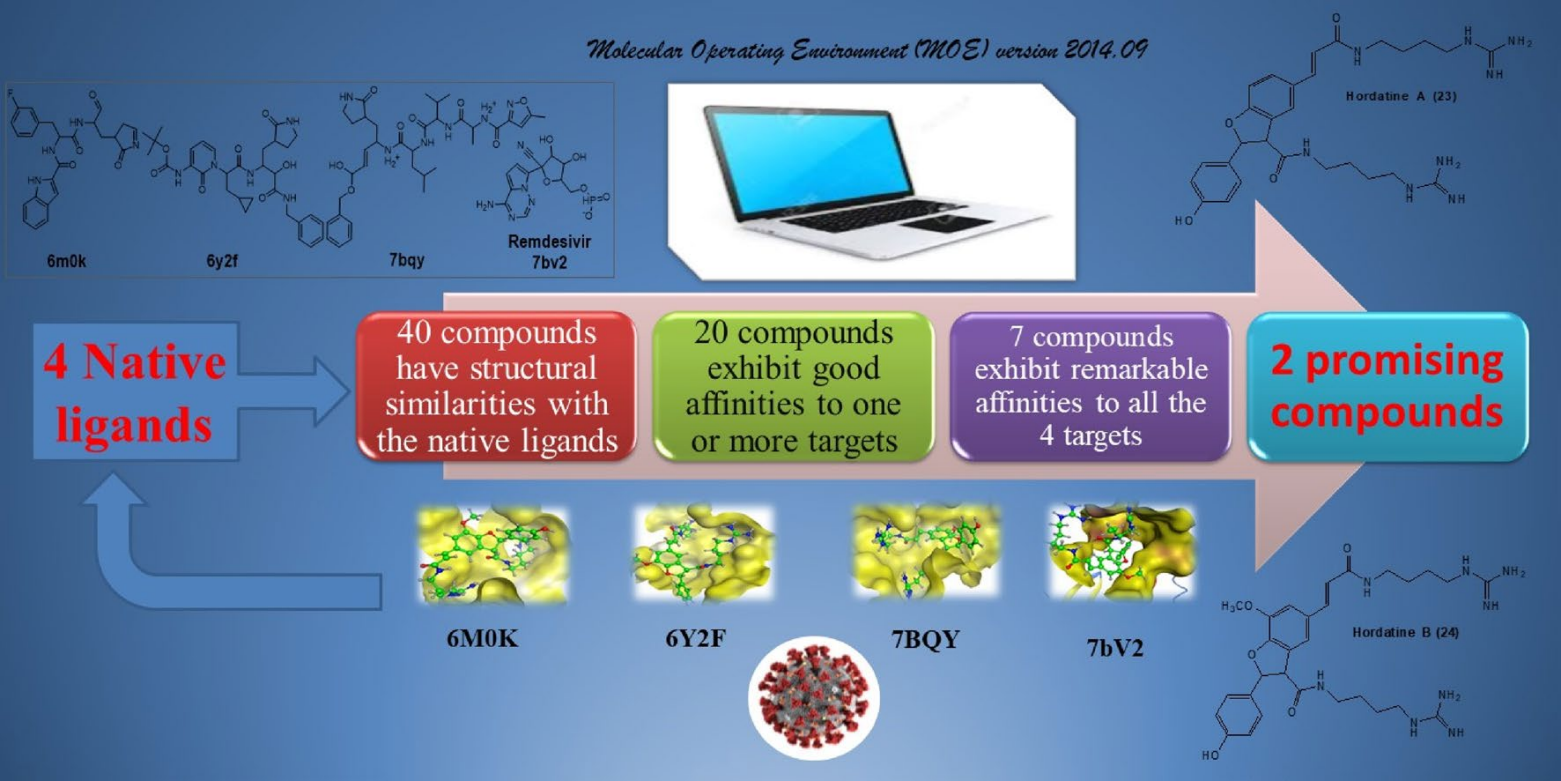

**Electronic supplementary material:**

The online version of this article (10.1007/s13659-020-00275-9) contains supplementary material, which is available to authorized users.

## Introduction

**Fig. 1:**
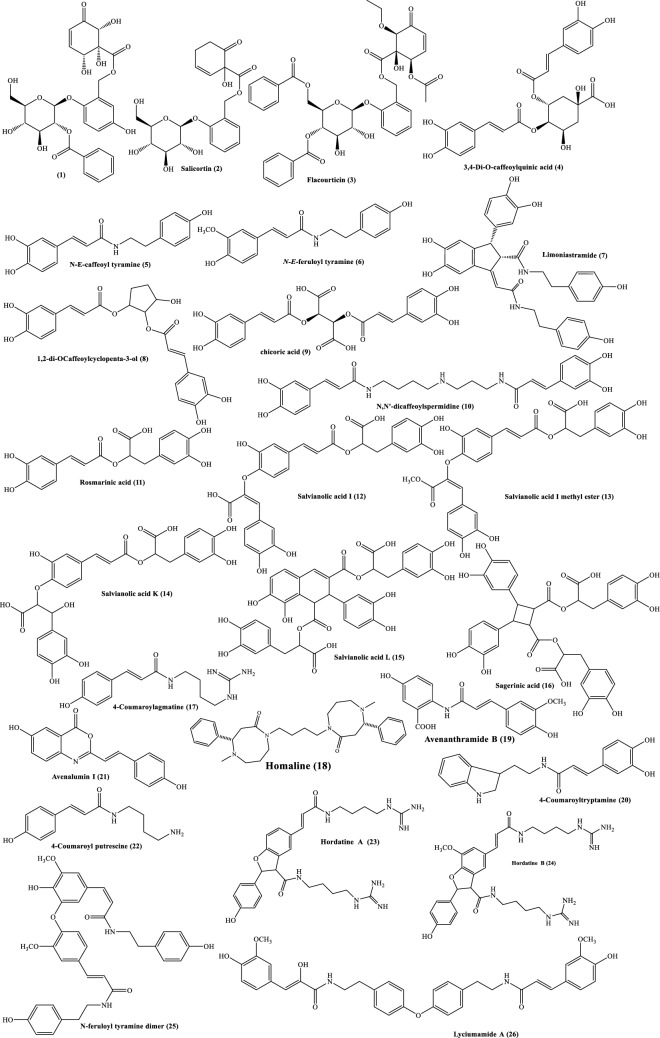

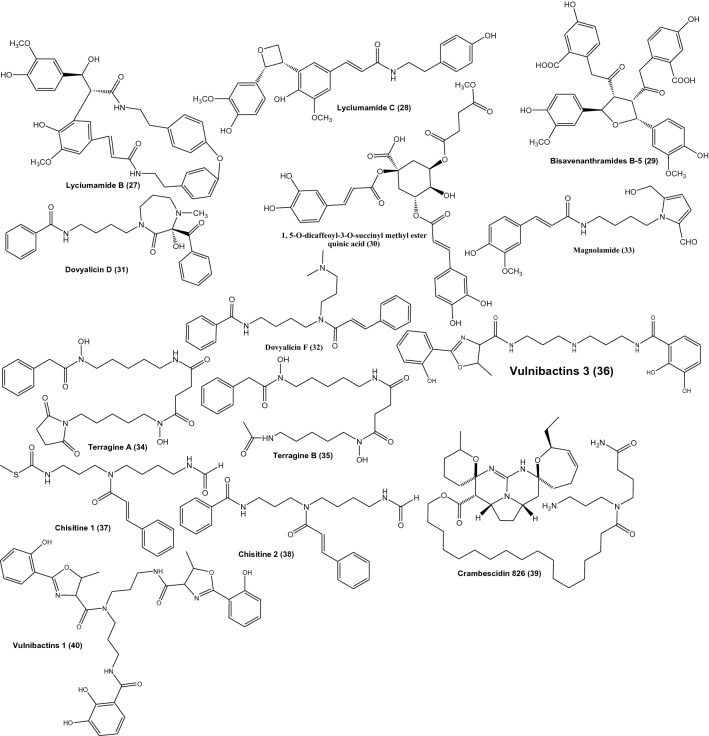
Structures of our target compounds

**Fig. 2:**
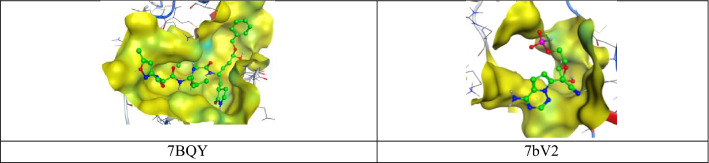
High-resolution crystal structures of coronavirus targets explain the native ligands in the active pockets (PDB: 7BQY and 7bV2)

**Fig. 3:**
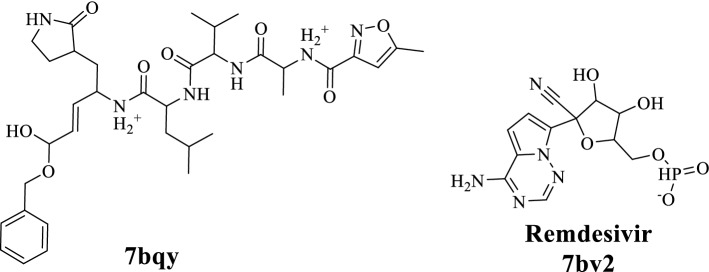
Structures of the native ligand of the target proteins (PDB ID: 7BQY and 7bV2)

During the last coronavirus outbreak, the rapid development of computer-aided drug discovery used for the in silico molecular modelling along with natural product databases have dramatically improved the drug development process. The inhibition of viral replication is a good strategy for antiviral drug discovery and development [1]. SARS-CoV replicase gene has been revealed to encode a number of enzymatic functions. These include RNA-dependent RNA polymerase (RdRp), 3C-like protease (3CLpro), a papain-like protease (PLpro) and a helicase. [2] 3C-like protease (3CLpro) and RNA polymerase play an important role in the replication of the virus and has a highly conserved catalytic domain from the SARS virus which is considered to be an attractive target for drug development [3]. By inhibiting anyone of these two proteins or both for a higher active therapy, the severity of the infection will be reduced. Natural products are inexhaustible source of drug discovery which always offers not only new compounds with interesting structures and different entities but also very important intermediates like shikimic acid which originated from *Illicium verum* fruits which serve as a source for synthesis and commercial production oseltamivir as an effective treatment for avian influenza virus H5N1, seasonal influenza virus types A and B and human influenza virus H1N1 of swine origin [4, 5]. Phenolic compounds and their derivatives are widely distributed in nature especially from plants, their diverse structures and combinations which may be founded as acids or esters or amides and also may including nitrogen in monomer or dimmer structures or even more [6]. Tens of phenolic compounds of different classes (phenolic acids, flavonoids and coumarins) are showing potent activities against many viruses like herpes simplex (HSV), influenza, epstein-barr hepatitis B and human immunodeficiency viruses (HIV) through different mechanisms [6, 7]. Antiviral activity and structure diversity and complexity of phenolic acids like caffeic acid derivatives as a major compounds of this study make them a suitable candidates for exploring their activity against COVID-19 [8]. Caffeic acid exhibit a potent antiviral activity against hepatitis C virus (HCV) at 55 nM level but its n-octyl ester derivatives showed a way more strongest anti-HCV activity at 1.0 to 109.6 picomolar level. The structure activity relationship revealed that n-alkyl side chain and catechol moiety are a pharmacophore responsible for the anti HCV activities [8, 9]. Chicoric acid or dicaffeoyltartaric acid is dimeric caffeic acid derivative with tartaric acid which also showed more potent antiviral activity against HIV [6]. Amide group is founded to increase antiviral activity of coumarin-based inhibitors against HIV through increasing biding affinity due hydrogen bonding [10]. A library of known 40 natural compounds (Fig. 1) have been run against the catalytic site of the COVID-19 main protease and RNA polymerase (Fig. 2). The selection of compounds was based on their structure similarities with COVID-19 main protease and RNA polymerase native ligands (Fig. 3). Hordatines, are dimers of coumaroylagmatine abundant in the shoots of barley seedlings grown in the dark, while no hordatines have been detected in un-germinated seeds or roots. Hordatine A is a dimer of coumaroylagmatine, while hordatine B possesses a methoxy group on its coumaran skeleton, is a dimer of coumaroylagmatine and feruloylagmatine [11, 12]. Hordatines may be stored as the glycosylated form in mature grains and partially produced by hydrolysis of the glycosylated form after germination explaining why hordatines have not previously been found in grains before germination [12]. The hordatines are antifungal substances, inhibit the spore germination of a number of fungi in concentrations as low as 10^–5^.[13] The concentrations of hordatines show maxima 6 days after germination and decline to less than 50% by the 11th day [11]. It has also been reported that Hordatine contents in barley leaves increase after an infection of powdery mildew [12]. Barley (*Hordeum vulgare* L.) Family; Poaceae was one of the first domesticated grains near the Nile river. It is used as animal fodder, source of fermentable material for distilled beverages, soups and stews food [14]. Barley has antiviral activities in addition to various properties, including; anti-inflammatory, antioxidant, diuretic, aphrodisiac, antiprotozoal, demulcent, astringent, febrifuge, digestive, expectorant, antimutagenic, refrigerant, sedative, stomachic, tonic properties, emollient, hypocholesterolemia effect, glycaemia regulation and wounds treatment [14, 15].

## Results, Discussion and Conclusion

**Fig. 4:**
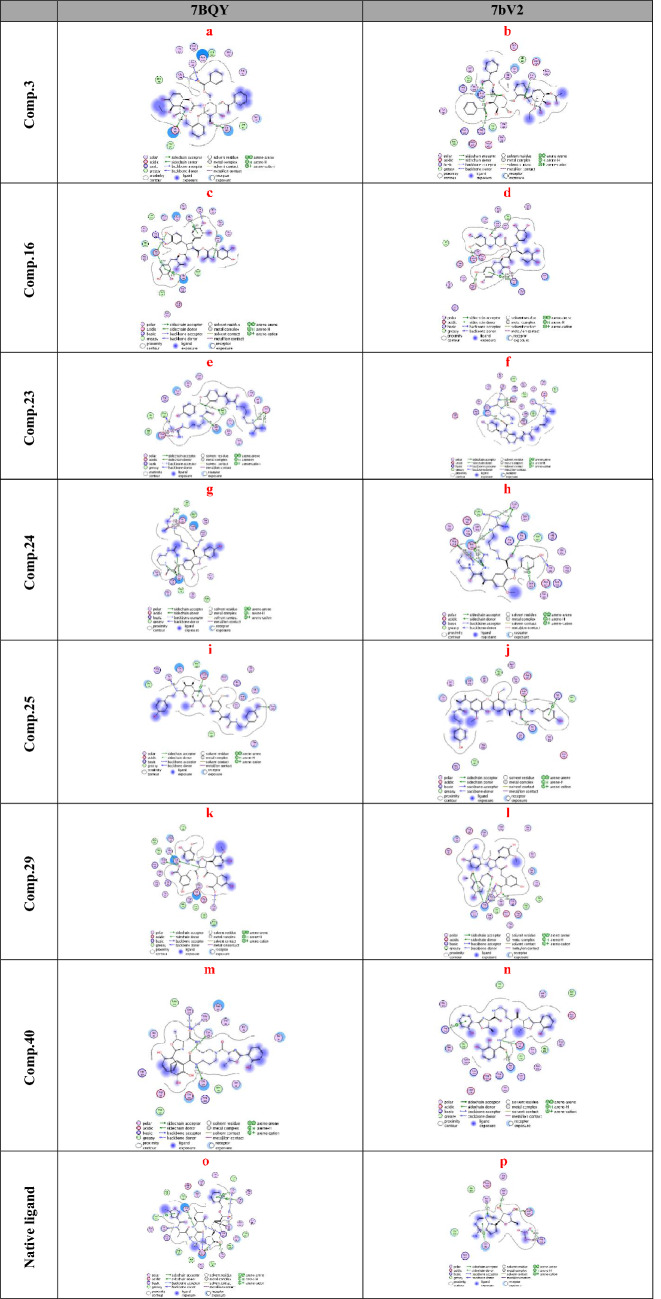
Best Molecular docking patterns of candidate compounds **3**, **16**, **23**, **24**, **25**, **29**, **40** and native ligands into 7BQY and 7bV2

Totally we docked 40 compounds to 4 COVID-19 targets (3 protease and 1 RNA polymerase). A comparative analysis can be done by referring to (Table 1). As for binding affinities, 20 compounds exhibit good binding affinities to one or more of the COVID-19 targets. Surprisingly, 7 out of 20 compounds exhibit remarkable binding affinities to all the 4 targets (6M0K, 6Y2F, 7BQY or 7bV2). The top 7 hits are flacourticin (**3)**, sagerinic acid (**16)**, hordatine A (**23)**, hordatine B (**24)**, *N*-feruloyl tyramine dimer (**25)**, bisavenanthramides B-5 (**29)** and vulnibactins (**40)** summarized in (Table 2). Docking interactions pattern of the top 7 hits are depicted in (Fig. 4). Importantly, hordatines (**23 and 24)** were found to interact with both protease and polymerase by exhibiting the highest binding affinity through forming strong hydrogen bonds with some residues of the catalytic site, as well as significant extra interactions with other receptor binding residues.

**Table 1:** Comparative docking study results on COVID-19 enzymes

Comp.	COVID-19 main drug targets	
	Main protease	RNA polymerase
	7BQY (resolution: 1.7)	7BV2 (resolution: 2.5)
**1**	−	−
**2**	−	−
**3**	+	+
**4**	−	−
**5**	−	−
**6**	−	−
**7**	+	−
**8**	−	−
**9**	−	−
**10**	−	−
**11**	−	−
**12**	−	−
**13**	−	+
**14**	−	−
**15**	+	+
**16**	+	+
**17**	−	−
**18**	−	−
**19**	−	−
**20**	−	−
**21**	−	−
**22**	−	−
**23**	+	+
**24**	+	+
**25**	+	+
**26**	−	+
**27**	+	+
**28**	+	−
**29**	+	+
**30**	+	+
**31**	−	−
**32**	−	−
**33**	−	−
**34**	−	−
**35**	−	−
**36**	−	+
**37**	−	−
**38**	−	−
**39**	+	+
**40**	+	+

For Comparative docking study results on protease targets 6M0K and 6Y2F see Supplementary Data

**−** Indicates that dock score value is higher than **−**7.5, **+** indicates that dock score value is **−**7.5 or lower

**Table 2:** MOE binding energies S (Kcal mol^−1^) of best binding pose for compounds 3, 16, 23, 24, 25, 29, 40 and native ligands into 7BQY and 7bV2 (London dG as score function)

Comp	Protein	Receptor	Distance (Å)	S (London dG)
**3**	7BQY	Glu166-Gln189-Gly143-Ser144	2.9, 2.9, 3.2, 3.2	− 8.3
	7BV2	Arg553-Arg553-Arg553-Arg553-Arg624-Ser759	3.1, 3.2, 3.2, 2.7, 3.2, 2.9	− 8.7
**16**	7BQY	Glu166-Glu166-His164-Gln189-Gly143-Thr26	3.3, 4.5, 3.1, 4.2, 3.7, 2.6	− 9.1
	7BV2	Arg553-Arg553-Arg553-Arg553-Arg553-Arg553-Asp623-Asp760-Ser814	2.9, 3.6, 3.8, 2.9, 3.0, 3.5, 3.1, 2.9, 2.8	− 8.5
**23**	7BQY	Glu166-Glu166-Glu166-Glu166-Glu166-phe140-Gln189-Met49-Glu47-Glu47-Glu47-Glu47-Glu47-Glu47	2.99-3.01-3.79-3.26-3.9-3.26-3.13-4.06-3.3-3.3-3.52-2.9-2.9-3.6	− 9.0
	7BV2	Arg553-Arg553-Thr680- Asp623-Asp623-Asp623-Asp623-Asp684-Ser682	2.97, 2.83, 3.19, 3.38, 3.38, 3.24, 3.24, 2.9, 2.87	− 8.11
**24**	7BQY	Glu166-Glu166-Glu166-Glu166-Glu166-Glu166-phe140-Gly143-Gln189-Gln189	3.4, 3.4, 3.2, 3.6, 3.9, 2.7, 3.0, 2.8, 3.2, 3.0	− 8.5
	7BV2	Thr680-Ser682-Arg553-Cys813-Cys813-Leu758-Asp618-Asp618-Asp618-Asp618-Asp760-Asp760-Asp760-Asp760-Asp760-Asp760-Asp760	3.1, 3.6, 3.6, 3.6, 3.6, 4.2, 2.9, 3.8, 3.5, 2.8, 2.8, 3.5, 3.5, 3.0, 3.9, 3.0, 2.9, 2.9	− 8.3
**25**	7BQY	Glu166-Thr190-Asn119	3.9, 2.9, 3.0	− 7.9
	7BV2	Asp760-Arg553-Arg553-Lys621	3.0, 2.8, 3.0, 3.8	− 8.2
**29**	7BQY	Glu166-Gln192-His41-Cys145-His41	2.8, 3.2, 3.1, 3.4, 3.1	− 7.7
	7BV2	Arg553-Arg553-Arg553-Arg553-Arg553-Arg553-Arg553-Arg624-Arg624	2.9, 2.9, 3.1, 3.2, 3.4, 3.7, 2.8, 2.9, 2.9	− 8.3
**40**	7BQY	Ser144-Gly143-Cys145-Gln189	3.3, 3.1, 3.5, 2.7	− 7.9
	7BV2	Arg553-Arg553-Asp760-Arg836	3.2, 3.0, 3.2, 4.1	− 8.2
Ligands	7BQY	Glu166-Glu166-Glu166-Gln189-His163-His164-Cys145-Gln189-Thr190-Ala191-Thr26-Thr25	3, 2.7, 2.6, 3.2, 2.5, 3.0, 3.1, 3.2	− 7.8
	7BV2	Arg553-Arg553-Arg553-Arg553-Arg553-Asp623-Asn691-Ser759-Ser682	2.3, 2.4, 2.3, 2.4, 3.9, 2.3, 2.7, 2.6, 4.1	− 5.9

### For Protease Target 7BQY

Binding interactions of the native ligand (binding score = − 7.8) (Fig. 4o**),** revealed that there are 3 hydrogen bonds with Glu166. In addition to other bonds with Gln189, His163, His164, Cys145, Gln189, Thr190, Ala191, Thr26 and Thr25. Whereas in case of hordatine A binding interactions with 7BQY (binding score = − 9.0) is given in (Fig. 4f)**,** five hydrogen bonds were recorded with Glu166. Furthermore, extra nine interactions were observed with phe140, Gln189, Met49 and Glu47. Whereas in case of hordatine B binding interactions with 7BQY (binding score = − 8.5) is given in (Fig. 4g), 6 bonds were recorded with Glu166. In addition to other interactions were observed with phe140, Gly143 and Gln189 (Table 2).

**Table 3:** MOE binding energies S (Kcal mol^−1^) of best binding pose for compounds **3**, **16**, **23**, **24**, **25**, **29**, **40** and native ligands into 7BQY and 7bV2 (ASE as score function)

Comp	Protein	S (ASE)
**3**	7BQY	− 30.5
	7BV2	− 31.7
**16**	7BQY	− 34.6
	7BV2	− 35.8
**23**	7BQY	− **30.1**
	7BV2	− **34.5**
**24**	7BQY	− **31.8**
	7BV2	− **30.6**
**25**	7BQY	− 35.9
	7BV2	− 27.9
**29**	7BQY	− 28.5
	7BV2	− 32.1
**40**	7BQY	− 30.3
	7BV2	− 28.8
Ligands	7BQY	− 32.9
	7BV2	31.8

For Molecular docking Patterns of Candidate Compounds **3**, **16**, **23**, **24**, **25**, **29** and **40** into Protease Targets 6M0K and 6Y2F See Supplementary Data.

### For Polymerase Target 7bv2

Binding interactions of the native ligand Remdesivir (binding score = − 5.9) (Fig. 4p), revealed that there are 5 hydrogen bonds with Arg553. In addition to other bonds with Asp623, Asn691, Ser759 and ser682. For hordatine A binding interactions with 7bv2 (binding score = − 8.11) is given in (Fig. 4h), two hydrogen bonds were recorded with Arg553. Furthermore, extra interactions were observed with Thr680, Asp623, Asp684 and Ser682. Whereas in case of hordatine B binding interactions with 7bv2 (binding score  = − 8.3) is given in (Fig. 4i), Hydrophobic interaction was recorded with Ser682 and one hydrogen bond was recorded with Arg553. In addition to seven interactions were recorded with Asp760. Furthermore, 4 interactions were recorded with Asp618. Also, other interactions were observed with thr680, cys813 and Leu7582. It is worth mentioning that remdesivir is a nucleotide analogue prodrug that inhibits viral RNA polymerases which has shown in vitro prophylactic and therapeutic efficacy in nonclinical models against COVID-19 [16].

Hordatines A and B could be obtained by extraction from dark-grown barley by several methods as a mixture. One method by homogenization with 2 volume acetic acid followed by centrifugation for 5 min. after being left at 4 °C for 20 h. The supernatant was decanted, and the precipitate washed with 1 volume acetic acid and re-centrifugated. Both acetic acid decanted fractions were combined and evaporated till dryness at 40 °C*.* The solid residue was re-dissolved in 1 volume 2–5% trichloroacetic acid and kept for 15 min. then centrifuged for 5 min. and the supernatant was stored at − 10 °C [11]. Another method was reported by shaking pearled grain flour with 1 mL of 75% acetone for 60 min. at room temperature, in three or four replications then centrifugate at 12,500 rpm for 10 min. followed by re-extracting the precipitate with 75% acetone twice. Both acetone extracts evaporated under vacuum and dissolved in 3 mL 2.5% acetic acid [12]. Another method was reported by sowing barley seeds in flats containing heat sterilized vermiculite. and incubated in the dark in controlled environment chambers. The developed shoots were extracted by boiling with 100 mL water for 10 min. The filtered extract was shaken with Amberlite IR C 50 (H^+^) ion-exchange resin (5 g dry weight) for 1 h. the supernatant liquid was decanted, and the resin rinsed with several portions of water. The adsorbed bases were then eluted by shaking the resin with 100 mL 2 N acetic acid for 1 h and filtering [13]. Hordatines A and B could be obtained by solid-phase extraction [12] or by cationic exchange resin using buffer (0.05 M NaCl/0.13 M NaOH), pH 13 at 95 °C in a mixture form. [17]. Hordatine A is probably synthesized by oxidative dimerization of coumaroylagmatine [11]. Hordatine B could be biosynthesized in two consecutive reactions. In the first, agmatine coumaroyltransferase (ACT) catalyzes the conjugation of agmatine and p-coumaroyl-CoA or feruloyl-CoA. In the second reaction peroxidase catalyzes the oxidative coupling of agmatine conjugates by linking coumaroylagmatine and feruloylagmatine [12]. Identification of hordatines A and B is achieved using TLC of Avicel (R_*f*_ 0.54 for A) and (R_*f*_ 0.53 for B) developed with the upper phase of n-butanol—water—acetic acid (4:5:1) in pre-saturated tanks. The Sakaguchi reagent (specific for guanidines), diazotized nitroaniline solution, and alcoholic bromocresol green were used as chromogenic sprays [13].

Since 40 natural compounds were subjected to virtual screening using two different molecular docking protocols against 3 protease and one RNA polymerase targets of COVID-19. The compounds exhibited variable degrees of affinities toward COVID-19 targets comparing to the native inhibitor. Seven compounds were found to interact with all COVID-19 targets by exhibiting the most acceptable binding affinity through forming strong hydrogen bond with the catalytic sites. For RNA polymerase target of COVID-19 (PDB ID: 7bV2), these seven compounds were found to have better binding scores than the native ligand, remdesivir, the well-known antiviral drug. Importantly, hordatine (**23** and **24**) phenolic compounds present in barley, were found to interact with both protease and polymerase by exhibiting the highest binding affinity through forming strong hydrogen bonds with the catalytic residues, as well as significant extra interactions with other receptor-binding residues. Such compounds are recommended to be tested clinically for proposed activity against COVID-19. They may be tested either alone or in combinations. In addition, our results may facilitate the future design and synthesis of new candidates against COVID-19.

## Experimental Section

### Literature Search and Compounds Selection

To find a natural inhibitor for COVID-19; search was conducted in the following databases: Science Direct, PubMed and Google Scholar for published articles. Selected compounds in this study were included based on structural similarities with the native ligands (Fig. 3) which contain terminal aromatic ring(s) and aliphatic chain with ester or amide groups. Forty compounds belong to phenolic amides, phenolic esters and amide alkaloids were selected.

### Molecular Docking Analysis

**Fig. 5:**
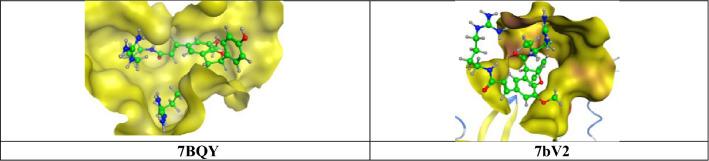
High-resolution crystal structures of compound **24** in the active pockets (PDB ID: 7BQY and 7bV2)

**Fig. 6:**
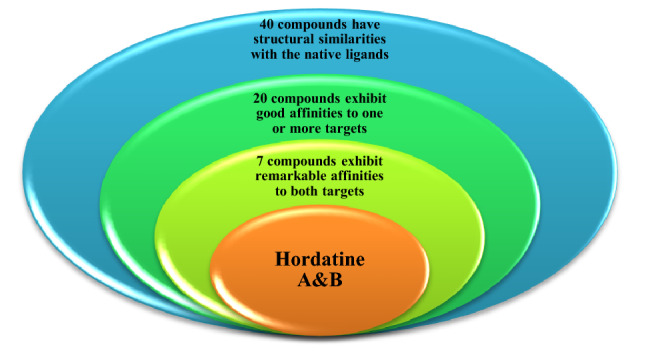
An outline of the employed virtual screening methodology

Molecular docking is a structure-based drug design approach to identify the essential amino acid interactions between the selected protein and generated ligands with low energy conformation.[18] Molecular operating environment MOE, package version 2014.09 software was used for computational analysis. The compound was subjected to 3D protonation and energy minimization up to 0.01 gradient. Crystal structures of (PDB IDs: 6M0K, 6Y2F, 7BQY and 7bV2) were selected and obtained from Protein Data Bank (https://www.rscb.org) with good resolutions [19–22]. The crystal structures were imported into MOE The structure preparation wizard of MOE was used to correct all the issues in protein structures. The hydrogen atoms were added to structures in their standard geometry, all solvent molecules were removed from the structures and then subjected to energy minimization. The final optimized structures were saved in the working directory. Triangle matcher and refinement methods were used for performing docking studies. We run two docking protocols with two different score functions, London dG (Table 2) and ASE (Table 3). The obtained compound–receptor complexes were then used to study the predicted ligand-receptor attachments at the target sites and their binding energies. In order to find a potential candidate for treating COVID-19, molecular docking studies were performed over 40 natural molecules on the binding pocket of COVID-19 enzymes (PDB IDs: 6M0K, 6Y2F, 7BQY and 7bV2) (Fig. 2). The list of drugs tested for docking study is depicted in (Fig. 1). All these 40 molecules were docked against the 4 targets and ranked based on their dock score. Compounds having dock score of − 7.5 or less are considered better agent for inhibition of the COVID-19 target (Figs. 5 and 6).

## Electronic supplementary material

Below is the link to the electronic supplementary material.

Supplementary file1 (DOCX 2987 kb)

## Data Availability

The docking results are available upon request from the Hatem S. Abbass.
