# Supplementary material for: Hordatines as a Potential Inhibitor of COVID-19 Main Protease and RNA Polymerase: An In-Silico Approach
Source: Nat Prod Bioprospect. 2020 Oct 22;10(6):453–62. doi: 10.1007/s13659-020-00275-9 (PMC7579552; doi:10.1007/s13659-020-00275-9)
Supplement: Supplementary file 1 — Supplementary file1 (DOCX 2987 kb) [file 13659_2020_275_MOESM1_ESM.docx]

**Hordatines as a Potential Inhibitor of COVID-19 Main Protease and RNA Polymerase: An In-Silico Approach**

**Mohammed A. Dahab^a^, Mostafa M. Hegazy^b^, Hatem S. Abbass^b,c*^**

1. Department of Pharmaceutical Medicinal Chemistry and Drug Design, Faculty of Pharmacy, Al-Azhar University (Boys), Cairo 11884-Egypt
2. Department of Pharmacognosy, Faculty of Pharmacy, Al-Azhar University (Boys), Cairo 11884-Egypt
3. Department of Pharmacognosy, Faculty of Pharmacy, Sinai University, Kantara 41636-Egypt

***Supplementary data***

**In sillico study**

**For protease target 6M0K**, Binding interactions of the native ligand (binding score = - 7.1) (Figure 3o), revealed that there are 2 hydrogen bonds with Glu166. In addition to other interactions with His 41, His 164, His 163 and Cys 145. Whereas in case of Hordatine A binding interactions with 6M0K (binding score = - 8.5) is given in (Figure 3e)**,** six hydrogen bonds were recorded with Glu166 which is assumed to be essential for the activity. Furthermore, extra interactions were observed with Phe144, Asn142 and Gln189. For Hordatine B Binding interactions with 6M0K (binding score = - 8.7) is given in (Figure 3g), six bonds were recorded with Glu166 which is assumed to be essential for the activity. In addition to a hydrophobic interaction was observed with His41. In addition to other interactions with Arg188, val186, Met165 and Asn142.

**For protease target 6Y2F**, Binding interactions of the native ligand (binding score = - 7.1) (Figure 3p)**,** revealed that there are 3 hydrogen bonds with Glu166. In addition to other bonds with His 41, His 164, His 163, Cys 145, Ser144 and Gly143. Whereas in case of Hordatine A binding interactions with 6M0K (binding score = - 8.0) is given in (Figure 3f)**,** three hydrogen bonds were recorded with Glu166. Furthermore, extra eight interactions were observed with ser144, Gln192, Glu47 and Met49. Whereas in case of Hordatine B Binding interactions with 6Y2F (binding score = - 8.5) is given in (Figure 3h), 6 bonds were recorded with Glu166. In addition to strong interactions are observed with phe140 and Thr26. Furthermore, Strong hydrogen bond was observed with Asn142.

| 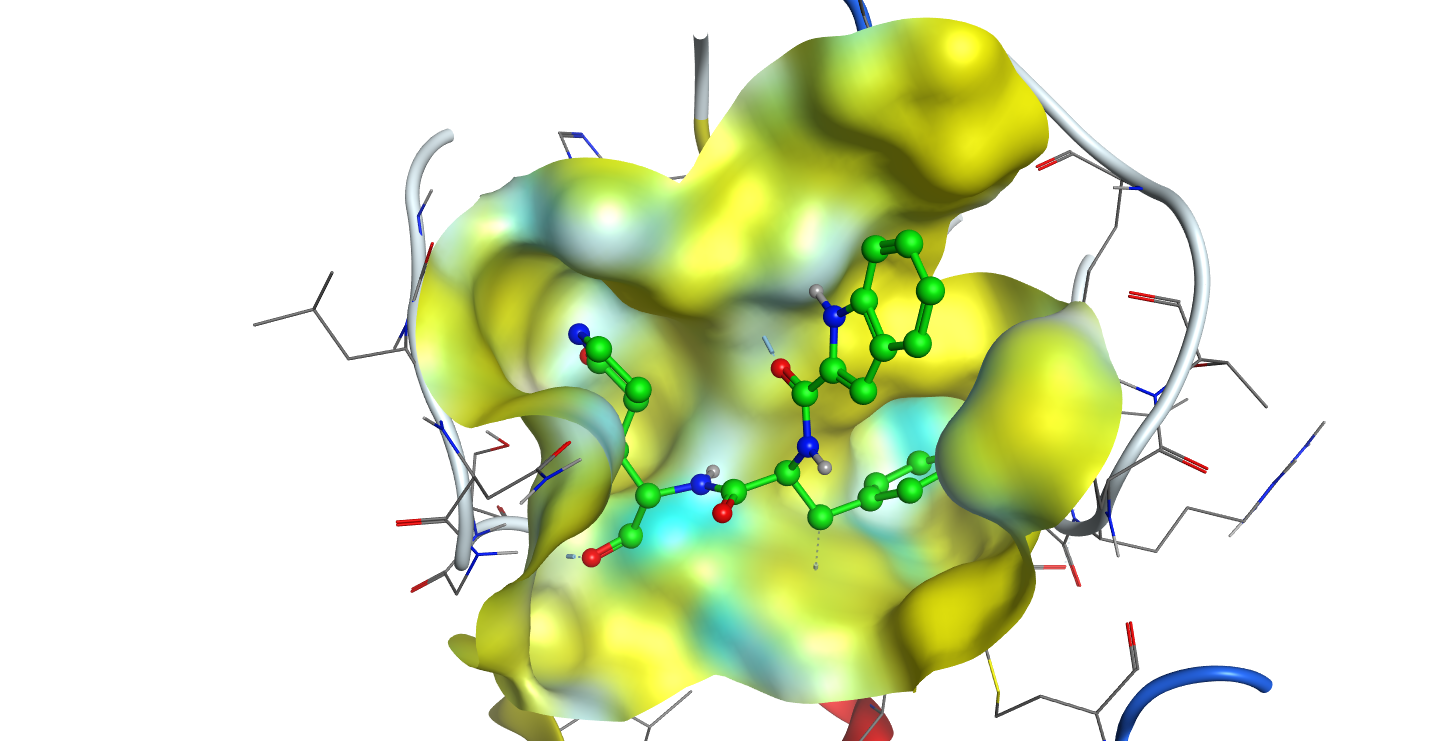6M0K | 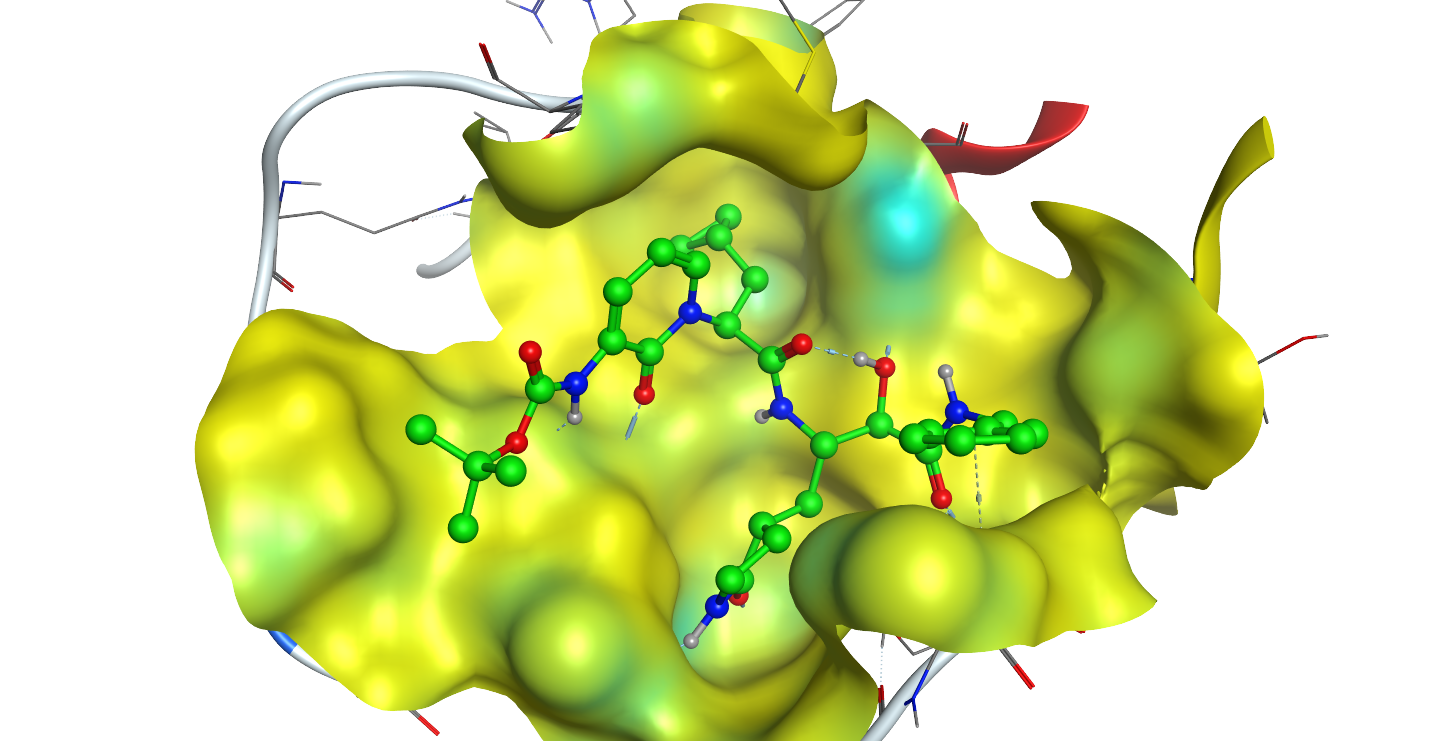6Y2F |
| --- | --- |
| **Figure 1: High-resolution crystal structures of coronavirus targets explain the native ligands in the active pockets (PDB ID: 6M0K and 6Y2F).** | |

|  |
| --- |
| **Figure 2: Structures of the native ligand of the target proteins (PDB ID: 6M0K and 6Y2F)** |

|  | **6M0K** | **6Y2F** |
| --- | --- | --- |
| **Comp.3** | a  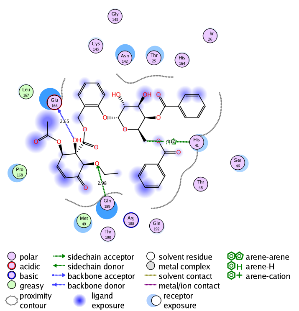 | b  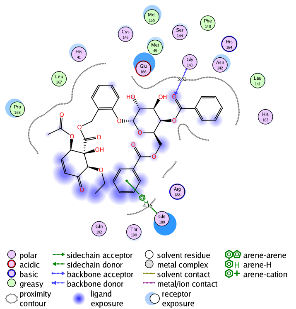 |
| **Comp.16** | c  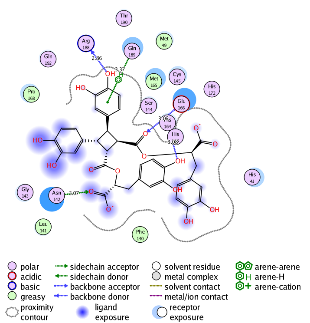 | d  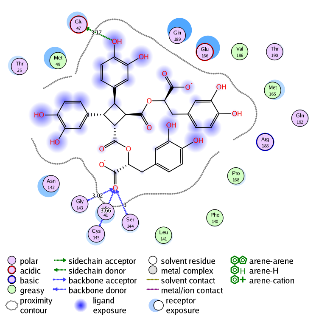 |
| **Comp.23** | e  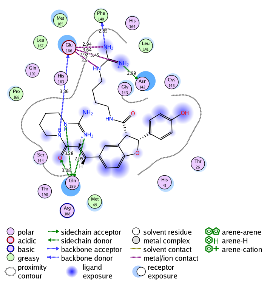 | f  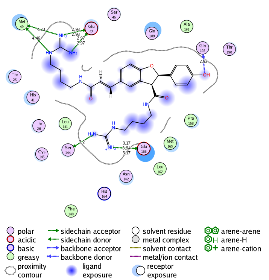 |
| **Comp.24** | g  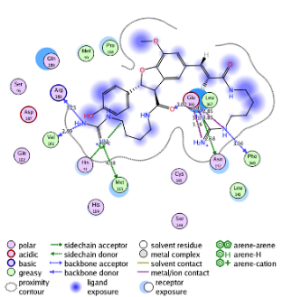 | h  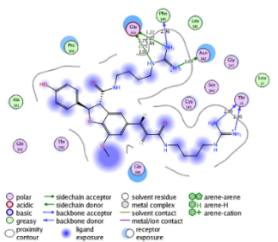 |
| **Comp.25** | i  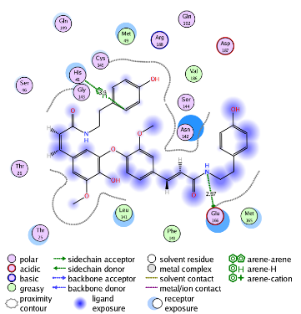 | j  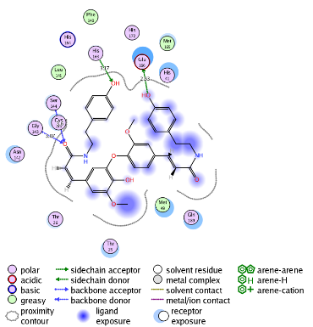 |
| **Comp.29** | k  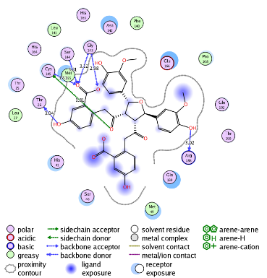 | l  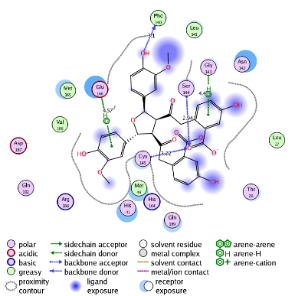 |
| **Comp.40** | m  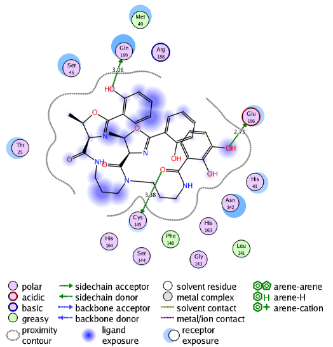 | n  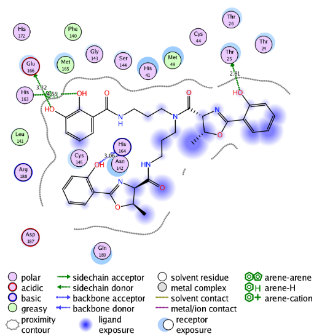 |
| **Native ligand** | o  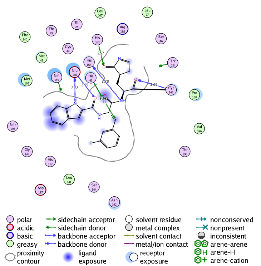 | p  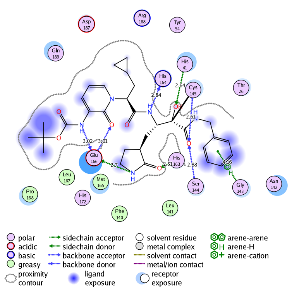 |
| **Figure 3: Best Molecular docking patterns of candidate compound 3, 16, 23, 24, 25, 29, 40 and native ligands into 6M0K and 6Y2F.** | | |

| 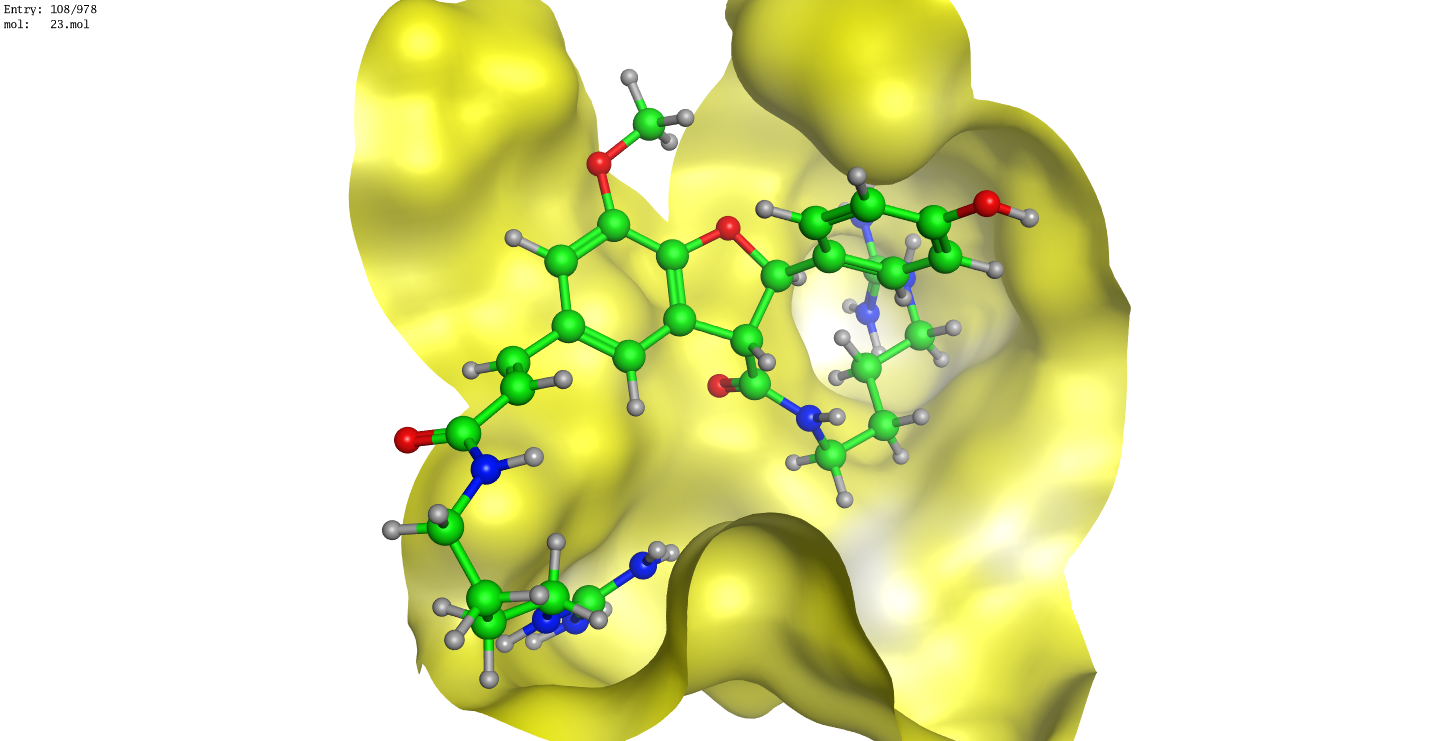6M0K | 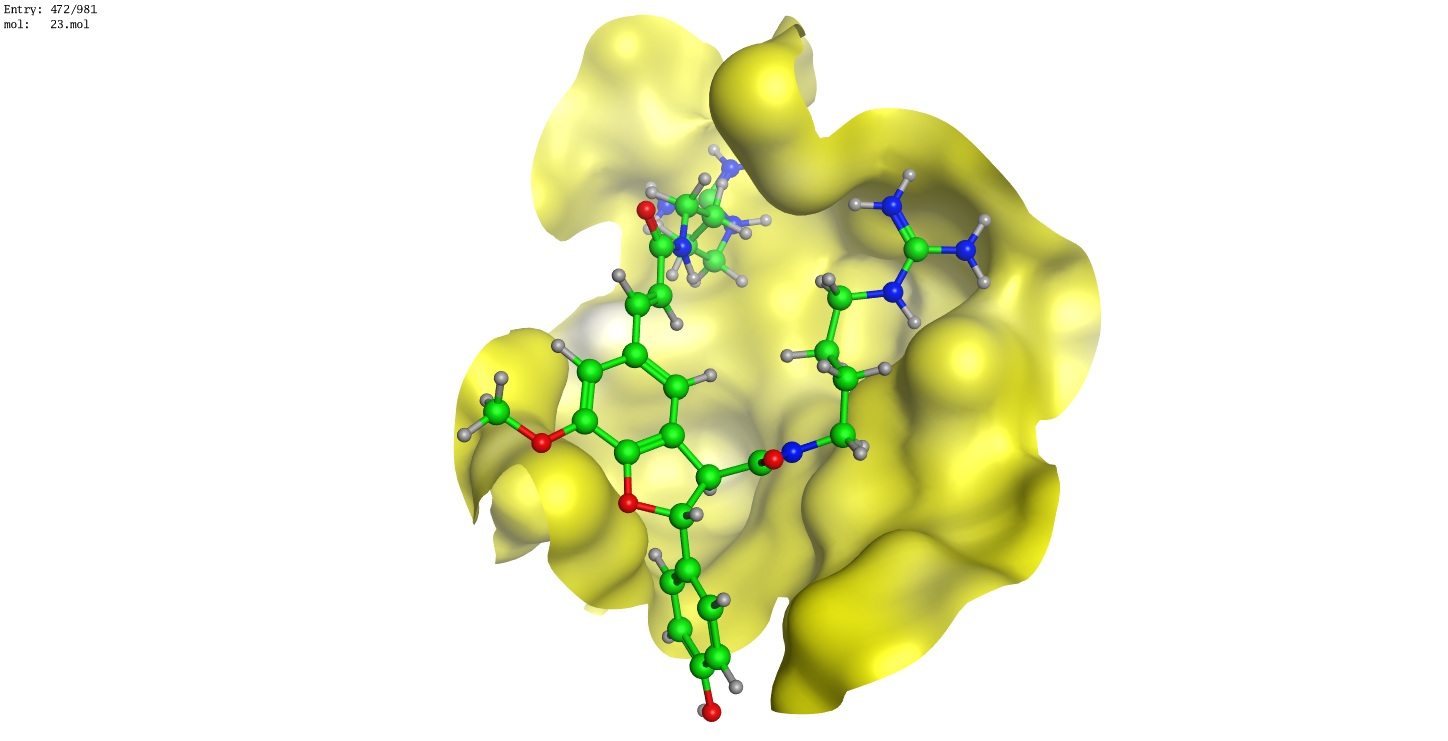6Y2F |
| --- | --- |
| **Figure 4: High-resolution crystal structures of compound 24 in the active pockets (PDB ID: 6M0K and 6Y2F).** | |

| **Table 1: Comparative docking study results on COVID-19 main Protease** | | |
| --- | --- | --- |
|  | 6M0K  (Resolution: 1.5) | 6Y2F  (Resolution: 1.9) |
| 1 | **-** | **-** |
| 2 | **-** | **-** |
| 3 | **+** | **+** |
| 4 | **-** | **+** |
| 5 | **-** | **-** |
| 6 | **-** | **-** |
| 7 | **-** | **+** |
| 8 | **-** | **+** |
| 9 | **-** | **+** |
| 10 | **-** | **-** |
| 11 | **-** | **-** |
| 12 | **-** | **-** |
| 13 | **+** | **-** |
| 14 | **-** | **+** |
| 15 | **-** | **+** |
| 16 | **+** | **+** |
| 17 | **-** | **-** |
| 18 | **-** | **-** |
| 19 | **-** | **-** |
| 20 | **-** | **-** |
| 21 | **-** | **-** |
| 22 | **-** | **-** |
| 23 | **+** | **+** |
| 24 | **+** | **+** |
| 25 | **+** | **+** |
| 26 | **+** | **+** |
| 27 | **+** | **-** |
| 28 | **-** | **-** |
| 29 | **+** | **+** |
| 30 | **-** | **-** |
| 31 | **-** | **-** |
| 32 | **-** | **-** |
| 33 | **-** | **-** |
| 34 | **-** | **-** |
| 35 | **-** | **-** |
| 36 | **-** | **-** |
| 37 | **-** | **-** |
| 38 | **+** | **-** |
| 39 | **+** | **-** |
| 40 | **+** | **+** |
| **-** Indicates that dock score value is higher than -7.5.  **+** Indicates that dock score value is -7.5 or lower. | | |

| **Table 2: MOE binding energies S (Kcal mol^-1^) of best binding pose for compounds 3, 16, 23, 24, 25, 29, 40 and native ligands into 6M0K and 6Y2F (London dG as score function).** | | | | |
| --- | --- | --- | --- | --- |
| **Comp.** | **Protein** | **Receptor** | **Distance (Å)** | **S (London dG)** |
| **3** | 6M0K | Glu166-His41-Gln189 | 2.7, 4.2, 2.9 | -7.6 |
|  | 6Y2F | Gln189-Gly143 | 4.2, 3.5 | -7.8 |
| **16** | 6M0K | Glu166-His163-Gln189-Arg188-Asn142 | 3.0, 3.0, 3.4, 2.9, 3.0 | -8.9 |
|  | 6Y2F | Glu47-Gly143-Cys145-Ser144 | 3.2, 3.0, 3.7, 3.0 | -8.0 |
| **23** | 6M0K | Glu166- Glu166- Glu166- Glu166- Glu166- Gln189- Gln189- Gln189- phe140- Asn142 | 2.94, 3.64, 3.45, 2.92, 3.5, 3.63, 3.08, 3.28, 2.95, 2.85, 2.99 | -8.5 |
|  | 6Y2F | Glu166- Glu166- Glu166- ser144- Gln192- Glu47- Glu47- Glu47- Glu47- Met49- Met49 | 3.17, 3.94, 3.17, 3.2, 2.92, 2.93, 2.93, 2.97, 2.97, 4.23, 4.46 | -8.0 |
| **24** | 6M0K | Glu166- Glu166- Glu166- Glu166- Glu166- Glu166-Asn142-Phe140-Arg188-Val186-His41-met165 | 3.0, 3.4, 3.2, 3.2, 2.9, 3.9, 2.7, 3.4, 3.3, 2.9, 4.0, 4.1 | -8.7 |
|  | 6Y2F | Glu166- Glu166- Glu166- Glu166- Glu166- Glu166-phe140-asn142-thr26- thr26 | 3.2, 3.2, 3.4, 2.7, 2.7, 3.9, 2.9, 3.0, 2.9, 2.9 | -8.5 |
| **25** | 6M0K | Glu166-His41 | 2.9, 3.9 | -7.8 |
|  | 6Y2F | Glu166-His163-Ser144-Gly143 | 2.9, 3.1, 3.6, 3.0 | -8.3 |
| **29** | 6M0K | Arg188-Thr26-Cys145-Cys145-Ser144-Gly143-Gly143 | 3.0, 3.0, 3.8, 3.5, 3.4, 3.1, 2.9 | -8.8 |
|  | 6Y2F | Glu166-Phe140-Gly143-Ser144-Cys145 | 4.5, 3.1, 4.4, 2.9, 3.2 | -8.7 |
| **40** | 6M0K | Gln189-Cys145-Glu166 | 3.0, 3.3, 2.7 | -8.1 |
|  | 6Y2F | Glu166-His153-His164-Thr25 | 3.3, 4.5, 3.0, 2.8 | -7.7 |
| **Ligands** | 6M0K | Glu166-Glu166-His41-His164-His163-Cys145 | 2.6, 2.8, 3.9, 2.8, 2.6, 2.7 | -7.1 |
|  | 6Y2F | Glu166- Glu166- Glu166-His41-His164-His163-Cys145-Ser144-Gly143 | 3.0, 3.0, 2.7, 2.6, 2.8, 2.5, 2.6, 2.9, 4.2 | -7.1 |

| **MOE binding energies S (Kcal mol^-1^) of best binding pose for the top hits and native ligands into main Protease 7BQY (ASE as scoring function).** |
| --- |
| 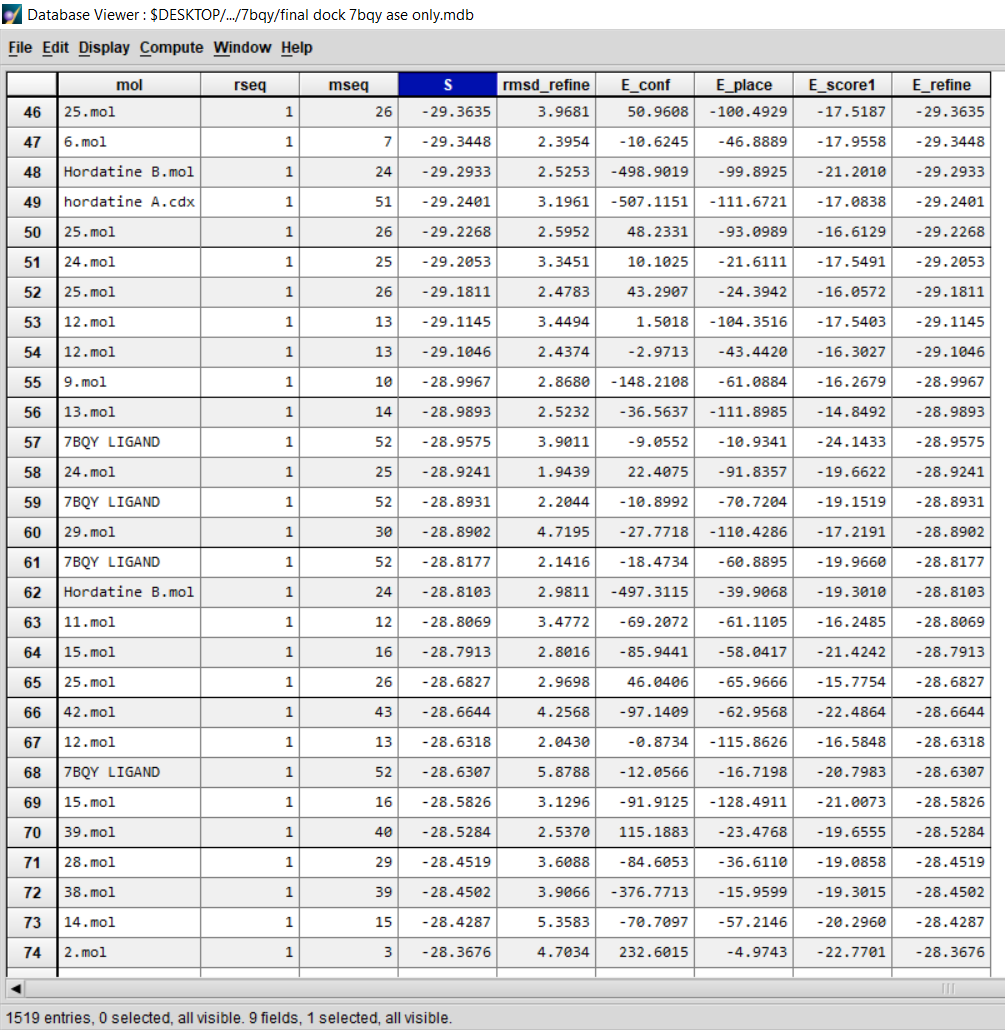 |
| **MOE binding energies S (Kcal mol^-1^) of best binding pose for the top hits and native ligand into RNA polymerase 7bV2 (ASE as scoring function).** |
| 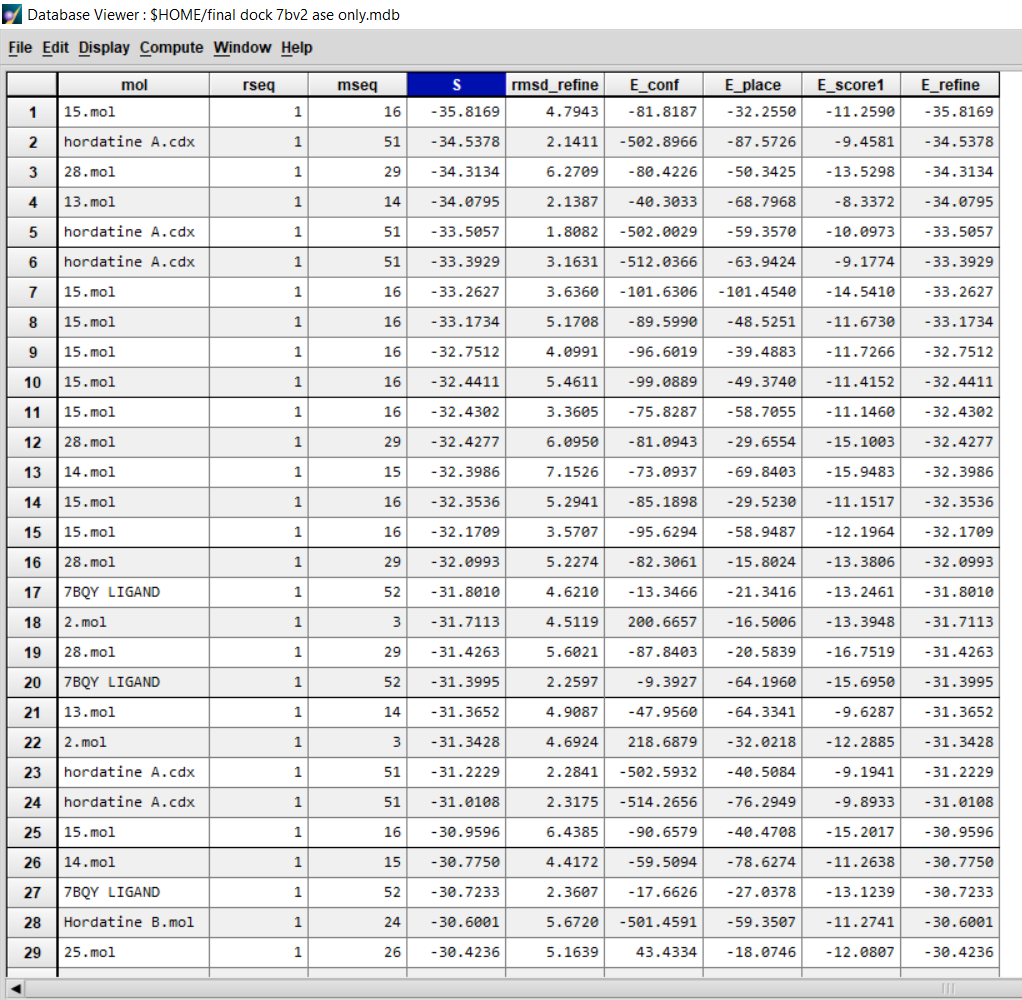 |
